# Relative Changes in Krill Abundance Inferred from Antarctic Fur Seal

**DOI:** 10.1371/journal.pone.0027331

**Published:** 2011-11-07

**Authors:** Tao Huang, Liguang Sun, John Stark, Yuhong Wang, Zhongqi Cheng, Qichao Yang, Song Sun

**Affiliations:** 1 Institute of Polar Environment, School of Earth and Space Sciences, University of Science and Technology of China, Hefei, China; 2 Department of Biology and the Ecology Center, Utah State University, Logan, Utah, United States of America; 3 Institute of Oceanology, Chinese Academy of Sciences, Qingdao, China; 4 Brooklyn College, Brooklyn, New York, United States of America; Institut Pluridisciplinaire Hubert Curien, France

## Abstract

Antarctic krill *Euphausia superba* is a predominant species in the Southern Ocean, it is very sensitive to climate change, and it supports large stocks of fishes, seabirds, seals and whales in Antarctic marine ecosystems. Modern krill stocks have been estimated directly by net hauls and acoustic surveys; the historical krill density especially the long-term one in the Southern Ocean, however, is unknown. Here we inferred the relative krill population changes along the West Antarctic Peninsula (WAP) over the 20th century from the trophic level change of Antarctic fur seal *Arctocephalus gazella* using stable carbon (δ^13^C) and nitrogen (δ^15^N) isotopes of archival seal hairs. Since Antarctic fur seals feed preferentially on krill, the variation of δ^15^N in seal hair indicates a change in the proportion of krill in the seal's diets and thus the krill availability in local seawater. For the past century, enriching fur seal δ^15^N values indicated decreasing krill availability. This is agreement with direct observation for the past ∼30 years and suggests that the recently documented decline in krill populations began in the early parts of the 20th century. This novel method makes it possible to infer past krill population changes from ancient tissues of krill predators.

## Introduction

Over the past 50 years, the West Antarctic Peninsula (WAP) has experienced rapid regional warming associated with significant sea-ice and krill stock reductions [1]–[3]. More importantly, Antarctic krill is a key species in Southern Ocean food webs that supports large amounts of fishes, seabirds and marine mammals in Antarctic marine ecosystems [4]–[6]. Environmental variability throughout the WAP could lead to cascading trophic level changes [7], particularly for krill predators such as the Antarctic fur seal. From a predator's perspective, trophic level and dietary change of krill predators is a reflection of relative krill population changes [8]. Therefore, past relative krill abundance, which is difficult to obtain, could be inferred from the paleodiet of krill predators.

Stable isotope analyses of animal tissues are increasingly recognized as a powerful tool for quantifying animal's foraging habitat and trophic level [9]. For example, stable carbon (δ^13^C) and nitrogen (δ^15^N) isotope signatures of hair and whisker have been used to infer animal diets [10], [11]. Keratinous tissues can preserve dietary information for long time periods [10], particularly in Antarctica, and archival keratinous hairs are ideal material for studying the dietary history of krill predators. Well-preserved hairs and droppings in lake sediments have also been used to infer past populations of seals and penguins [12]–[14].

In this study, we propose a novel stable isotope methodology for deducing long-term relative krill population dynamics based upon the trophic level of krill predators, and inferred the relative krill population change of the 20th century using the δ^15^N of Antarctic fur seal hair. Since Antarctic fur seals feed preferentially on krill [15], and their trophic level (indicated by δ^15^N) is controlled by their dietary compositions. Therefore, a shift in δ^15^N of seal hair indicates a change in the proportion of krill in the seal's diet, and such a change should be a reflection of the availability of krill in local seawater.

## Materials and Methods

### Ethics statement

The authors have declared that there is no ethics problem because our samples were extracted from lake sediment.

### Sampling area and chronology

**Figure 1:**
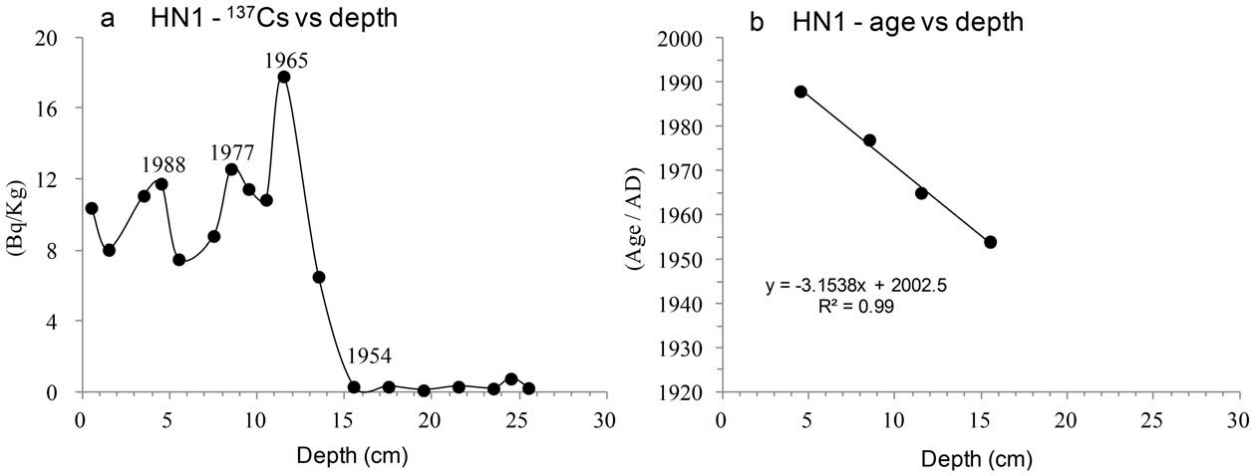
Chronology of HN1 by ^137^Cs dating. (a), ^137^Cs signal in the sediments of HN1. (b), ^137^Cs determined age versus depths in HN1.

Field study was carried out on Fildes Peninsula, King George Island, South Shetland Islands (62°02′S, 58°21′W). The sediment core HN1, 35.5 cm long, was retrieved from a lake catchment near a large fur seal colony. Usually, there are many female fur seals and only one male seal in the colony in summer, and seal remains such as excrements and hairs were shed to the lake and deposited into the sediments. The top 25.5-cm layer of HN1 contains seal excrement and seal hairs and is identified as seal excrement deposition; the section below 25.5 cm is littoral deposition with black basaltic sand. Here we focus on the top 25.5 cm. The chronology of HN1 was established by ^137^Cs dating on sediments, which was verified as a reliable dating method in previous studies [12], [16]. Based on the ^137^Cs signal in sediments, we determined the depth of 15.5 cm corresponds to 1954 AD, the beginning of the ^137^Cs sedimentation. The ^137^Cs peaks at depths of 11.5, 8.5, and 4.5 cm correspond to 1965 AD, 1977 AD and 1988 AD, respectively (Fig 1a). The bottom (25.5 cm) of the seal excrement deposition section was inferred corresponds to the early 20th century (approximately 1924 AD), based on the average sedimentation rate in the top 15.5 cm of the core (Fig 1b). These results are described in Yang et al. (2010) [17]. The hairs in the present study from the same sections were assumed to be from the same date.

### Stable isotope analyses

HN1 was sectioned at 0.5-cm intervals. Seal hairs were hand picked, cleaned using 2∶1 chloroform: methanol solution, dried at 40°C, and then weighed accurately into tin capsules. Seal hairs were analyzed for δ^13^C and δ^15^N by continuous-flow direct combustion and mass spectrometry using a Europa 20/20 SL isotope-ratio mass spectrometer at Utah State University. Precision is ±0.15‰ for δ^15^N and ±0.10‰ for δ^13^C. δ^15^N in sediments were determined by Finnigan-MAT-251 mass spectrometer at Institute of Soil Science, Chinese Academy of Sciences, and the precision is ±0.20‰. Results are presented in δ (‰) and expressed relative to air for δ^15^N and Vienna Pee Dee Belemnite (VPDB) for δ^13^C according to the equation: δ (‰) = [(R_sample_−R_standard_)/R_standard_] ×10^3^, where δ (‰) represents the δ^15^N or δ^13^C value, R_sample_ is the isotopic ratio of the sample, and R_standard_ of the air and VPDB.

### Statistical analyses

Regression analysis and t-test for the null hypothesis of a zero slope were performed on the time-series data of isotope values (n = 50) obtained from sediments and seal hairs in HN1 using SPSS 16.0.

## Results

**Figure 2:**
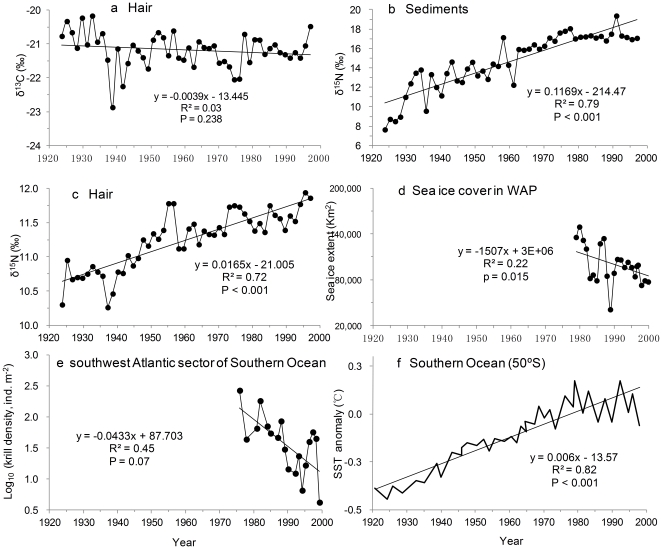
Changes in stable isotope values of seal hair in HN1 and regional biological and physical conditions. (a), δ^13^C values of seal hair. (b), δ^15^N values of seal excrement sediment. (c), δ^15^N values of seal hair. (d), Sea ice cover along the WAP [2]. (e), Krill populations change along the WAP [3]. (f), Southern Ocean SST anomalies [25].

The δ^13^C values of seal hairs range from −22.87‰ to −20.17‰ with a mean of −21.18‰ (Fig 2a) and show equivocal trends (t =  −1.196, p = 0.238). The δ^15^N values of seal excrement sediments range from 7.64‰ to 19.37‰ with a mean of 14.67‰ (Fig 2b). The δ^15^N values of seal hairs range from 10.26‰ to 11.94‰ with a mean of 11.24‰ (Fig 2c); There is an obvious difference in the rates of change between 1924–1957 and after 1957, that is the amplitude of variation in hair δ^15^N in 1924–1957 (10.30‰–11.78‰, a 1.48‰ difference) is larger than that in 1958–1997 (11.12‰–11.94‰, a 0.82‰ difference). The δ^15^N values of sediments are as enriched as 19.37‰, exceeding the normal δ^15^N values of marine mammals; the abnormally enriched δ^15^N values of sediments could be the result of large fractionation effects during ammonia volatilization from seal excrement [18]. The δ^15^N signature in hair, however, is not subject to this fractionation effect because hair is keratinized. The δ^15^N signatures of both sediments and hairs become significantly enriched over time (sediments: t = 13.47, p<0.001; hairs: t = 11.04, p<0.001). The 1.68‰ enrichment of δ^15^N in seal hairs indicates an obvious change of fur seal diets over time.

## Discussion

Due to the different metabolic rates of various tissues, stable isotope values reflect trophic levels at different time scales, from days for plasma and excrement to weeks and months for feathers and hairs [9], [19]. So the stable isotope values in archival hairs in HN1 reflect the average diets of fur seals during summer in their breeding seasons. In the Southern Ocean, δ^13^C values vary with latitudes and along an inshore/offshore gradient, and typical values for Antarctica fur seal are −23‰ ∼ −19‰ in Antarctica and −19‰ ∼ −16‰ in subantarctica [11]. The depleted δ^13^C values in HN1 clearly indicate foraging of fur seal in Antarctica during breeding seasons in the study area although we cannot rule out possible inshore/offshore influence.

Fecal analysis showed that Antarctic fur seals in South Shetland Islands feed mainly on krill and various fish species [20]. We estimated the range of δ^15^N values in seal hair with various percentages of krill vs. fish in seal diet using endmember bulk nitrogen isotope values, and the result shows that seal δ^15^N becomes enriched as krill proportion in seal diet decreases (Table 1). Krill availability affects predator's foraging behavior. In Scotia Sea, when krill is abundant, seals prey primarily on krill; and when krill is scarce, seals feed on krill and fish [15]. In Ross Sea, Adélie penguins eat more fish when krill availability is low, and vice versa [21]. The recent significant depletion of δ^15^N in Adélie penguin is ascribed to the ‘krill surplus’ in the Southern Ocean [22]. Dietary change of predators reflects relative abundance of their prey items. Therefore, the δ^15^N signature in seal hairs is linked to and thus could be used to infer krill availability and population.

**Table 1:** Estimated δ^15^N values in seal hair with various percentages of prey items in seal diets.

δ^15^N (‰) in prey/predator					%		of		prey		in		diet					
*Electrona antarctica* (8.90)*Gymnoscopelus nicholsi* (10.20)	1000	0100		0		2070		3333		1050		1040	1030	030		020		
*Krill* (5.20)	0	0		100		10		33		40		50	60	70		80		
*Seal hair*	11.90	13.20		8.20		12.44		11.10		11.07		10.57	10.07	9.70		9.20		

Note: The δ^15^N of hairs are added 3.0‰ for fractionation effect according to Hobson et al. [28]. Prey δ^15^N values are compiled from Emslie & Patterson [22] for krill and Cherel et al. [29] for *Electrona antarctica* and *Gymnoscopelus nicholsi*.

The significantly enriching trend of the δ^15^N signature in HN1 for the last century indicates rising seal trophic levels and decreasing proportion of krill in seal diets, and this strongly suggests that local krill populations could have been in decline since the early 20th century. Two independent evidences support this inference. First, although there are no complete krill density databases for the past century, the decreasing krill stock in this region since 1970 s is well documented [3] and consistent with our results in overall trend. Second, krill abundance is closely linked with the sea ice extent and duration [3], [23], [24]. In this region for the past decades, the sea ice shows a decline trend [2] (Fig 2d), and this is in coincidence with the decline trend in krill populations [3] (Fig 2e). Like the seal δ^15^N values, the sea surface temperature (SST) anomaly in Southern Ocean (50°S) also shows an obvious increasing trend for the 20th century [25] (Fig 2f), and the significant correlation between them (r^2^ = 0.82, p<0.001) suggests that the inferred decreasing krill population is linked with warming ocean and declining sea ice extent.

Two other plausible explanations for the enriching δ^15^N values in the sediment core HN1 could be excluded. First, an enriching baseline (δ^15^N of the primary producer) may have contributed to the enriching seal δ^15^N values. The δ^15^N values of the Southern Ocean primary producer (diatom) are enriched during cold periods and depleted during warm periods [26]. Over the last 50 years, the present study site experienced a rapid air and ocean warming [1], [27], and the δ^15^N values of local primary producer were expected to be depleted. Therefore, the enriching baseline explanation seems unlikely. Second, an increasing intra-specific competition could also have led to the rising trophic level of seal in this study. The seal populations inferred from HN1 show rapid increase between 1955 and 1965 due to the ban of sealing [17], but the seal δ^15^N values do not show corresponding ‘rapid’ enrichment, indicating that the intra-specific competition is not responsible for seal dietary changes. Or it is also possible that the increases in seal populations might still have been too low during that time for krill stocks to be limited. Thus, the rising fur seal trophic levels are caused mainly by reduced krill availability and dietary changes.

Krill is the major consumer of diatom primary producers, the major food source for upper trophic predators, and the major fishery resource in the Southern Ocean. Krill density is of critical importance for the apex predator, and its massive biomass underpins the entire Southern Ocean ecosystems. Therefore finding a potential method for inferring krill populations change would be highly beneficial for research and management purposes. We could provide only a single core (HN1) for stable isotope analyses at present due to the limits of logistics. Further core analysis in other areas in Antarctica could be performed in future by international collaborations in field. Nevertheless, the results from HN1 do show that δ^15^N values in Antarctic fur seal hair can be used as an indicator for seal trophic level changes and the potential relative krill population dynamics. And long-term δ^15^N series from krill predators could provide vital information about the relative krill abundance in the Holocece epoch and its responses to abrupt or transitional climatic and environmental changes.
